# Bis[μ-4-(4-carb­oxy­phen­oxy)phthalato]bis­[triaqua­cobalt(II)]

**DOI:** 10.1107/S1600536813000536

**Published:** 2013-01-12

**Authors:** Liang Wang

**Affiliations:** a Department of Chemistry, University of Science and Technology Beijing, Beijing 100083, People’s Republic of China

## Abstract

The dinuclear title complex, [Co_2_(C_15_H_8_O_7_)_2_(H_2_O)_6_], lies across an inversion center. The unique Co^II^ ion is coordinated in a slightly distorted octa­hedral coordination geometry by two O atoms from a chelating 4-(carb­oxy­phen­oxy)phthalate ligand, three water O atoms and a further O atom from a bridging carboxyl­ate group of a symmetry-related 4-(carb­oxy­phen­oxy)phthalate ligand. In the crystal, O—H⋯O hydrogen bonds link the mol­ecules into a three-dimensional network.

## Related literature

For background to metal-organic coordination complexes, see: Wang *et al.* (2009 ▶); Leininger *et al.* (2000 ▶). For Co—O bond lengths in related structures, see: Chu *et al.* (2011 ▶). For the isotypic Ni^II^ complex and the synthesis, see: Cai (2011 ▶).
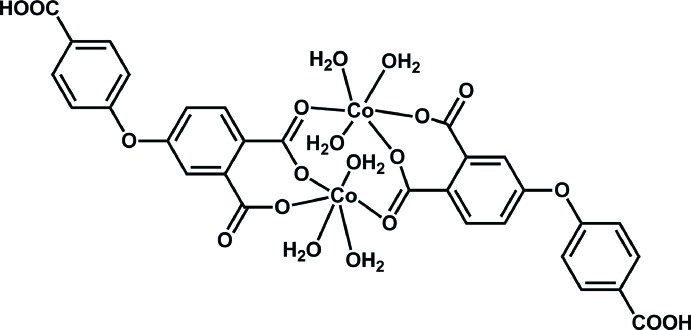


## Experimental

### 

#### Crystal data

[Co_2_(C_15_H_8_O_7_)_2_(H_2_O)_6_]
*M* *_r_* = 826.38
Monoclinic, 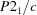

*a* = 14.451 (11) Å
*b* = 9.558 (7) Å
*c* = 11.404 (9) Å
β = 92.749 (15)°
*V* = 1573 (2) Å^3^
*Z* = 2
Mo *K*α radiation
μ = 1.15 mm^−1^
*T* = 293 K
0.15 × 0.12 × 0.10 mm

#### Data collection

Bruker APEXII diffractometer
Absorption correction: multi-scan (*SADABS*; Sheldrick, 2003 ▶) *T*_min_ = 0.847, *T*_max_ = 0.894
8135 measured reflections
3087 independent reflections
1591 reflections with *I* > 2σ(*I*)
*R*_int_ = 0.115

#### Refinement

*R*[*F* ^2^ > 2σ(*F* ^2^)] = 0.059
*wR*(*F* ^2^) = 0.116
*S* = 0.90
3087 reflections
235 parameters
9 restraints
H-atom parameters constrained
Δρ_max_ = 0.41 e Å^−3^
Δρ_min_ = −0.61 e Å^−3^

### 

Data collection: *APEX2* (Bruker, 2004 ▶); cell refinement: *SAINT* (Bruker, 2001 ▶); data reduction: *SAINT*; program(s) used to solve structure: *SHELXS97* (Sheldrick, 2008 ▶); program(s) used to refine structure: *SHELXL97* (Sheldrick, 2008 ▶); molecular graphics: *PLATON* (Spek, 2009 ▶); software used to prepare material for publication: *SHELXL97*.

## Supplementary Material

Crystal structure: contains datablock(s) global, I. DOI: 10.1107/S1600536813000536/lh5557sup1.cif

Structure factors: contains datablock(s) I. DOI: 10.1107/S1600536813000536/lh5557Isup2.hkl

Additional supplementary materials:  crystallographic information; 3D view; checkCIF report

## Figures and Tables

**Table 1:** Hydrogen-bond geometry (Å, °)

*D*—H⋯*A*	*D*—H	H⋯*A*	*D*⋯*A*	*D*—H⋯*A*
O8—H8*B*⋯O1^i^	0.85	2.06	2.839 (5)	152
O6—H6*A*⋯O2^ii^	0.85	1.77	2.598 (5)	165
O8—H8*A*⋯O7^iii^	0.84	2.14	2.865 (6)	144
O9—H9*A*⋯O3^iv^	0.85	2.06	2.861 (5)	157
O9—H9*B*⋯O7^v^	0.85	1.93	2.754 (5)	163
O10—H10*A*⋯O2^vi^	0.85	2.10	2.788 (5)	138
O10—H10*B*⋯O3^vii^	0.85	1.96	2.746 (5)	155

Symmetry codes: (i) 
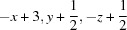
; (ii) 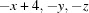; (iii) 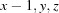; (iv) 
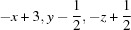
; (v) 
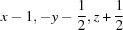
; (vi) 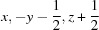; (vii) 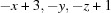.

**Fig. 1.**
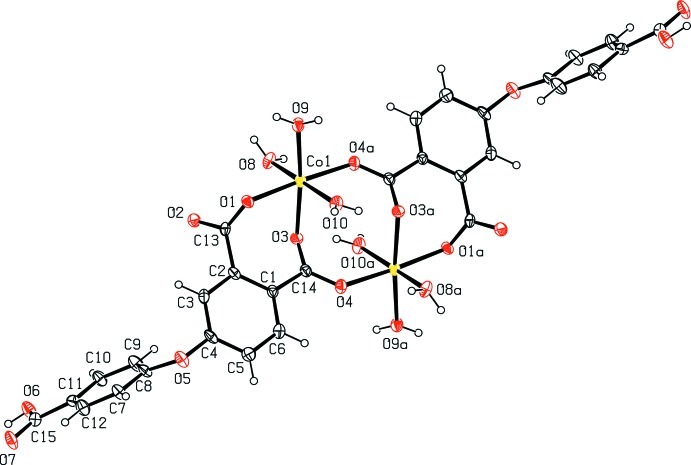
The molecular structure with displacement ellipsoids drawn at the 30% probability level, hydrogen atoms are omited for clarity [Symmetry code (a): -x+3, -y, -z+1].

**Fig. 2.**
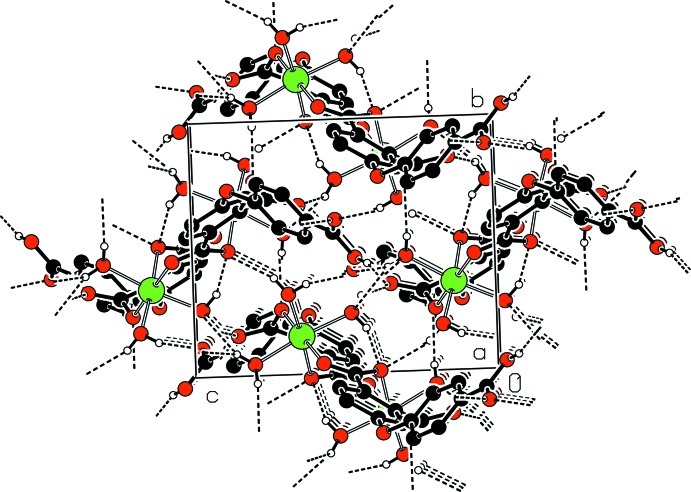
Part of the crystal structure with hydrogen bonds shown as dashed lines.

## supplementary crystallographic information

### Comment

In the field of supramolecular chemistry and crystal engineering, the design and assembly of metal-organic coordination complexes with appealing structures and properties have stimulated interests of chemists in recent decades (Wang *et al.*, 2009; Leininger *et al.* 2000)). Thus far, a large number of metal-organic coordination complexes have been fabricated. In this paper paper, the synthesis and crystal structure of the title compound, based on the multidentate 4-(4-carboxyphenoxy)phthalate ligand (H_3_L) is presented.

The molecular structure of the title compound is shown in Fig. 1. The dinuclear complex lies across an inversion center. The unique Co^II^ ion is coordinated in a slightly distorted octahedral coordination geometry by two oxygen atoms from a chelating 4-(carboxyphenoxy)phthalate ligand, three oxygen atoms from aqua ligands and a further O atom from a bridging carboxylate group of a symmetry related 4-(carboxyphenoxy)phthalate ligand. The Co—O bond lengths are as expected based on a a reported structure (Chu *et al.*, 2011). In the crystal, O—H···O hydrogen bonds link molecules into a three-dimensional network (Table 1 and Fig. 2). The crystal structure of the isostructural Ni(II) complex has been published (Cai, 2011).

### Experimental

The title compound was synthesized referring to a reported literature (Cai, 2011). H_3_L (0.030 g, 0.1 mmol), Co(OAc)_2_.4H_2_O (0.050 g, 0.2 mmol), and H_2_O (15 ml) was sealed in 25 ml Teflon-lined stainless steel reactor and heated to 393K. Purple blocks suitable for X-ray diffraction analysis were separated by filtration with the yield of 27%.

### Refinement

All H atoms bonded to C atoms were placed in geometrically idealized positions and treated as riding on their parent atoms with C—H = 0.93 Å, *U*_iso_ = 1.2U_eq_ (C). The hydrogen atoms of carboxyl group and water molecules were included in 'as found' positions and with O—H distances subsequently fixed at 0.85 (1)Å and U_iso_(H) = 1.5U_eq_(O).

### Crystal data

**Table:**

[Co_2_(C_15_H_8_O_7_)_2_(H_2_O)_6_]	*F*(000) = 844
*M**_r_* = 826.38	*D*_x_ = 1.744 Mg m^−^^3^
Monoclinic, *P*2_1_/*c*	Mo *K*α radiation, λ = 0.71073 Å
Hall symbol: -P 2ybc	Cell parameters from 679 reflections
*a* = 14.451 (11) Å	θ = 2.8–26.7°
*b* = 9.558 (7) Å	µ = 1.15 mm^−^^1^
*c* = 11.404 (9) Å	*T* = 293 K
β = 92.749 (15)°	Block, purple
*V* = 1573 (2) Å^3^	0.15 × 0.12 × 0.10 mm
*Z* = 2	

### Data collection

**Table:**

Bruker APEXII diffractometer	3087 independent reflections
Radiation source: fine-focus sealed tube	1591 reflections with *I* > 2σ(*I*)
Graphite monochromator	*R*_int_ = 0.115
φ and ω scans	θ_max_ = 26.0°, θ_min_ = 2.6°
Absorption correction: multi-scan (*SADABS*; Sheldrick, 2003)	*h* = −12→17
*T*_min_ = 0.847, *T*_max_ = 0.894	*k* = −11→11
8135 measured reflections	*l* = −13→14

### Refinement

**Table:**

Refinement on *F*^2^	Primary atom site location: structure-invariant direct methods
Least-squares matrix: full	Secondary atom site location: difference Fourier map
*R*[*F*^2^ > 2σ(*F*^2^)] = 0.059	Hydrogen site location: inferred from neighbouring sites
*wR*(*F*^2^) = 0.116	H-atom parameters constrained
*S* = 0.90	*w* = 1/[σ^2^(*F*_o_^2^) + (0.0393*P*)^2^] where *P* = (*F*_o_^2^ + 2*F*_c_^2^)/3
3087 reflections	(Δ/σ)_max_ = 0.001
235 parameters	Δρ_max_ = 0.41 e Å^−^^3^
9 restraints	Δρ_min_ = −0.61 e Å^−^^3^

### Special details

**Table:**

Geometry. All esds (except the esd in the dihedral angle between two l.s. planes) are estimated using the full covariance matrix. The cell esds are taken into account individually in the estimation of esds in distances, angles and torsion angles; correlations between esds in cell parameters are only used when they are defined by crystal symmetry. An approximate (isotropic) treatment of cell esds is used for estimating esds involving l.s. planes.
Refinement. Refinement of F^2^ against ALL reflections. The weighted R-factor wR and goodness of fit S are based on F^2^, conventional R-factors R are based on F, with F set to zero for negative F^2^. The threshold expression of F^2^ > 2sigma(F^2^) is used only for calculating R-factors(gt) etc. and is not relevant to the choice of reflections for refinement. R-factors based on F^2^ are statistically about twice as large as those based on F, and R- factors based on ALL data will be even larger.

### Fractional atomic coordinates and isotropic or equivalent isotropic displacement parameters (Å^2^)

**Table:**

	*x*	*y*	*z*	*U*_iso_*/*U*_eq_	
C1	1.7277 (4)	−0.0681 (5)	0.4420 (5)	0.0259 (13)	
C2	1.7404 (3)	−0.1470 (5)	0.3394 (4)	0.0278 (12)	
C3	1.8284 (4)	−0.1974 (5)	0.3174 (5)	0.0304 (14)	
H3	1.8371	−0.2486	0.2495	0.036*	
C4	1.9030 (4)	−0.1720 (6)	0.3955 (5)	0.0325 (14)	
C5	1.8912 (4)	−0.0994 (5)	0.4983 (5)	0.0379 (15)	
H5	1.9407	−0.0852	0.5521	0.045*	
C6	1.8029 (4)	−0.0477 (6)	0.5196 (5)	0.0377 (15)	
H6	1.7946	0.0021	0.5884	0.045*	
C7	2.1261 (4)	−0.2540 (6)	0.2792 (5)	0.0383 (15)	
H7	2.1418	−0.3268	0.3305	0.046*	
C8	2.0429 (4)	−0.1863 (5)	0.2862 (5)	0.0309 (14)	
C9	2.0170 (4)	−0.0799 (5)	0.2099 (5)	0.0392 (15)	
H9	1.9603	−0.0350	0.2153	0.047*	
C10	2.0772 (4)	−0.0415 (6)	0.1251 (5)	0.0361 (15)	
H10	2.0598	0.0288	0.0722	0.043*	
C11	2.1623 (4)	−0.1044 (5)	0.1169 (5)	0.0299 (13)	
C12	2.1871 (4)	−0.2131 (6)	0.1944 (5)	0.0392 (15)	
H12	2.2439	−0.2579	0.1893	0.047*	
C13	1.6620 (4)	−0.1895 (5)	0.2550 (5)	0.0263 (13)	
C14	1.6385 (4)	0.0012 (5)	0.4680 (5)	0.0271 (13)	
C15	2.2308 (4)	−0.0583 (6)	0.0309 (5)	0.0346 (14)	
O1	1.5943 (2)	−0.2597 (3)	0.2936 (3)	0.0293 (9)	
O2	1.6686 (2)	−0.1614 (4)	0.1480 (3)	0.0396 (10)	
O3	1.5760 (2)	0.0121 (3)	0.3846 (3)	0.0296 (9)	
O4	1.6298 (2)	0.0468 (3)	0.5707 (3)	0.0319 (9)	
O5	1.9905 (2)	−0.2297 (4)	0.3788 (3)	0.0389 (10)	
O6	2.1982 (3)	0.0438 (4)	−0.0387 (3)	0.0469 (11)	
H6A	2.2434	0.0675	−0.0795	0.070*	
O7	2.3071 (3)	−0.1081 (4)	0.0223 (3)	0.0488 (12)	
O8	1.4474 (2)	−0.0499 (3)	0.2018 (3)	0.0369 (10)	
H8A	1.4251	−0.0932	0.1424	0.055*	
H8B	1.4165	0.0250	0.2083	0.055*	
O9	1.3925 (2)	−0.3232 (3)	0.3192 (3)	0.0335 (9)	
H9A	1.4080	−0.3880	0.2729	0.050*	
H9B	1.3696	−0.3617	0.3786	0.050*	
O10	1.5100 (2)	−0.2415 (3)	0.5250 (3)	0.0353 (10)	
H10A	1.5400	−0.3082	0.5583	0.053*	
H10B	1.4919	−0.1802	0.5722	0.053*	
Co1	1.47938 (5)	−0.15569 (7)	0.35848 (6)	0.0266 (2)	

### Atomic displacement parameters (Å^2^)

**Table:**

	*U*^11^	*U*^22^	*U*^33^	*U*^12^	*U*^13^	*U*^23^
C1	0.028 (3)	0.023 (3)	0.027 (3)	−0.003 (3)	0.008 (3)	−0.001 (2)
C2	0.021 (3)	0.032 (3)	0.031 (3)	−0.001 (3)	0.006 (2)	0.004 (3)
C3	0.036 (4)	0.028 (3)	0.028 (3)	−0.001 (3)	0.012 (3)	−0.003 (2)
C4	0.022 (3)	0.038 (3)	0.038 (4)	−0.004 (3)	0.010 (3)	0.014 (3)
C5	0.037 (4)	0.042 (3)	0.035 (4)	−0.005 (3)	0.004 (3)	0.000 (3)
C6	0.037 (4)	0.042 (4)	0.034 (4)	0.003 (3)	0.008 (3)	−0.008 (3)
C7	0.026 (3)	0.041 (4)	0.048 (4)	0.003 (3)	0.010 (3)	0.010 (3)
C8	0.021 (3)	0.035 (3)	0.037 (3)	−0.005 (3)	0.007 (2)	0.006 (3)
C9	0.026 (3)	0.034 (3)	0.058 (4)	0.012 (3)	0.008 (3)	0.013 (3)
C10	0.031 (4)	0.038 (3)	0.040 (4)	0.001 (3)	0.008 (3)	0.014 (3)
C11	0.026 (3)	0.031 (3)	0.033 (3)	−0.004 (3)	0.001 (3)	−0.002 (3)
C12	0.029 (4)	0.040 (3)	0.049 (4)	0.014 (3)	0.007 (3)	0.001 (3)
C13	0.031 (3)	0.024 (3)	0.025 (3)	0.004 (2)	0.007 (3)	−0.004 (2)
C14	0.026 (3)	0.025 (3)	0.031 (4)	−0.003 (3)	0.008 (3)	−0.002 (3)
C15	0.033 (4)	0.041 (4)	0.030 (4)	−0.001 (3)	0.005 (3)	−0.005 (3)
O1	0.027 (2)	0.0238 (19)	0.038 (2)	−0.0057 (17)	0.0099 (17)	−0.0047 (17)
O2	0.036 (2)	0.055 (2)	0.028 (2)	−0.013 (2)	0.0070 (17)	0.000 (2)
O3	0.028 (2)	0.027 (2)	0.033 (2)	−0.0039 (18)	0.0031 (18)	−0.0051 (17)
O4	0.033 (2)	0.034 (2)	0.029 (2)	0.0054 (18)	0.0064 (17)	−0.0037 (18)
O5	0.029 (2)	0.046 (2)	0.043 (3)	0.007 (2)	0.0102 (19)	0.0128 (19)
O6	0.037 (3)	0.052 (3)	0.052 (3)	−0.001 (2)	0.018 (2)	0.015 (2)
O7	0.031 (2)	0.070 (3)	0.047 (3)	0.014 (2)	0.018 (2)	0.009 (2)
O8	0.053 (3)	0.031 (2)	0.027 (2)	0.0061 (19)	0.0038 (18)	−0.0044 (17)
O9	0.040 (2)	0.026 (2)	0.036 (2)	−0.0057 (18)	0.0120 (17)	−0.0065 (17)
O10	0.047 (3)	0.031 (2)	0.028 (2)	0.0081 (19)	0.0044 (18)	0.0052 (17)
Co1	0.0294 (4)	0.0233 (4)	0.0278 (4)	−0.0011 (4)	0.0072 (3)	−0.0018 (4)

### Geometric parameters (Å, º)

**Table:**

C1—C6	1.382 (7)	C11—C15	1.492 (7)
C1—C2	1.412 (7)	C12—H12	0.9300
C1—C14	1.491 (7)	C13—O2	1.258 (6)
C2—C3	1.394 (6)	C13—O1	1.282 (5)
C2—C13	1.506 (7)	C14—O4	1.262 (6)
C3—C4	1.387 (7)	C14—O3	1.283 (6)
C3—H3	0.9300	C15—O7	1.210 (6)
C4—C5	1.379 (7)	C15—O6	1.330 (6)
C4—O5	1.401 (6)	O1—Co1	2.101 (3)
C5—C6	1.402 (7)	O3—Co1	2.138 (3)
C5—H5	0.9300	O4—Co1^i^	2.085 (3)
C6—H6	0.9300	O6—H6A	0.8506
C7—C8	1.372 (7)	O8—Co1	2.086 (4)
C7—C12	1.395 (7)	O8—H8A	0.8445
C7—H7	0.9300	O8—H8B	0.8482
C8—C9	1.378 (7)	O9—Co1	2.071 (3)
C8—O5	1.392 (6)	O9—H9A	0.8509
C9—C10	1.381 (7)	O9—H9B	0.8511
C9—H9	0.9300	O10—Co1	2.096 (4)
C10—C11	1.376 (7)	O10—H10A	0.8500
C10—H10	0.9300	O10—H10B	0.8453
C11—C12	1.399 (7)	Co1—O4^i^	2.085 (3)
			
C6—C1—C2	118.4 (5)	O2—C13—C2	118.2 (5)
C6—C1—C14	118.1 (5)	O1—C13—C2	119.0 (5)
C2—C1—C14	123.4 (5)	O4—C14—O3	124.2 (5)
C3—C2—C1	119.4 (5)	O4—C14—C1	117.6 (5)
C3—C2—C13	117.1 (5)	O3—C14—C1	118.2 (5)
C1—C2—C13	123.3 (4)	O7—C15—O6	122.5 (5)
C4—C3—C2	120.8 (5)	O7—C15—C11	125.0 (6)
C4—C3—H3	119.6	O6—C15—C11	112.5 (5)
C2—C3—H3	119.6	C13—O1—Co1	120.2 (3)
C5—C4—C3	120.7 (5)	C14—O3—Co1	118.3 (3)
C5—C4—O5	117.5 (5)	C14—O4—Co1^i^	130.0 (4)
C3—C4—O5	121.5 (5)	C8—O5—C4	120.8 (4)
C4—C5—C6	118.4 (5)	C15—O6—H6A	105.3
C4—C5—H5	120.8	Co1—O8—H8A	120.7
C6—C5—H5	120.8	Co1—O8—H8B	115.5
C1—C6—C5	122.3 (5)	H8A—O8—H8B	107.6
C1—C6—H6	118.9	Co1—O9—H9A	121.4
C5—C6—H6	118.9	Co1—O9—H9B	114.6
C8—C7—C12	119.5 (5)	H9A—O9—H9B	107.6
C8—C7—H7	120.2	Co1—O10—H10A	141.2
C12—C7—H7	120.2	Co1—O10—H10B	104.3
C7—C8—C9	121.5 (5)	H10A—O10—H10B	113.7
C7—C8—O5	114.5 (5)	O9—Co1—O4^i^	90.40 (14)
C9—C8—O5	124.0 (5)	O9—Co1—O8	94.71 (14)
C8—C9—C10	118.5 (5)	O4^i^—Co1—O8	87.08 (14)
C8—C9—H9	120.7	O9—Co1—O10	89.59 (13)
C10—C9—H9	120.7	O4^i^—Co1—O10	88.57 (14)
C11—C10—C9	121.8 (5)	O8—Co1—O10	173.90 (14)
C11—C10—H10	119.1	O9—Co1—O1	92.25 (14)
C9—C10—H10	119.1	O4^i^—Co1—O1	176.92 (15)
C10—C11—C12	118.9 (5)	O8—Co1—O1	94.26 (14)
C10—C11—C15	122.6 (5)	O10—Co1—O1	89.89 (14)
C12—C11—C15	118.5 (5)	O9—Co1—O3	174.56 (13)
C7—C12—C11	119.7 (5)	O4^i^—Co1—O3	94.21 (14)
C7—C12—H12	120.2	O8—Co1—O3	82.66 (14)
C11—C12—H12	120.2	O10—Co1—O3	93.40 (14)
O2—C13—O1	122.6 (5)	O1—Co1—O3	83.22 (14)

Symmetry code: (i) −*x*+3, −*y*, −*z*+1.

### Hydrogen-bond geometry (Å, º)

**Table:**

*D*—H···*A*	*D*—H	H···*A*	*D*···*A*	*D*—H···*A*
O8—H8*B*···O1^ii^	0.85	2.06	2.839 (5)	152
O6—H6*A*···O2^iii^	0.85	1.77	2.598 (5)	165
O8—H8*A*···O7^iv^	0.84	2.14	2.865 (6)	144
O9—H9*A*···O3^v^	0.85	2.06	2.861 (5)	157
O9—H9*B*···O7^vi^	0.85	1.93	2.754 (5)	163
O10—H10*A*···O2^vii^	0.85	2.10	2.788 (5)	138
O10—H10*B*···O3^i^	0.85	1.96	2.746 (5)	155

Symmetry codes: (i) −*x*+3, −*y*, −*z*+1; (ii) −*x*+3, *y*+1/2, −*z*+1/2; (iii) −*x*+4, −*y*, −*z*; (iv) *x*−1, *y*, *z*; (v) −*x*+3, *y*−1/2, −*z*+1/2; (vi) *x*−1, −*y*−1/2, *z*+1/2; (vii) *x*, −*y*−1/2, *z*+1/2.
